# DNA barcode approaches to reveal interspecies genetic variation of Indian ungulates

**DOI:** 10.1080/23802359.2020.1719912

**Published:** 2020-01-29

**Authors:** Ranjana Bhaskar, Praveen Kanaparthi, Rengasamy Sakthivel

**Affiliations:** Southern Regional Centre, Zoological Survey of India, Chennai, India

**Keywords:** DNA barcoding, conservation, phylogeny, mammals, clade

## Abstract

In the past two decades, identification of species from noninvasive sampling has turned out to be an important tool for wildlife conservation. In this study a total 93 specimens representing 22 species of ungulates were analyzed from partial sequences of mtDNA *COI* and *Cytb* genes. All the species showed unique clades, and sequences divergence within species was between 0.01–3.9% in *COI* and 0.01–13.7 in *Cytb*, whereas divergence between species ranged from 2.2 to 29.5% in COI and 2.3 to 28.8% in Cytb. Highest intraspecific divergence was observed within the *Ovis aries* in COI and *Porcula salvania* in *Cytb*. Bayesian (BA) phylogeny analysis of both genes combined distinguishes all the studied species as monophyletic criteria. The Indian rhinoceros (*Rhinoceros unicornis*) exhibited closer relation to horse (*Equus caballus*). No barcode gap was observed between species in *COI*. This study demonstrates that even short fragments of *COI* and *Cytb* generated from fecal pellets can efficiently identify the Indian ungulates, thus demonstrating its high potential for use in wildlife conservation activities.

## Introduction

Ungulates are amongst the most vulnerable group of mammals (Ceballos et al. 2005). These are also known as the hoofed animals’ distinction due to the shape of their toe. Cattle, sheep, goats, deer and pigs belong to family Artiodactyla, horses and rhinos are part of another family Perissodactyla. There are 39 species of ungulates present in India (Sankar and Goyal 2004). Among these, many species are in the extremely endangered category (Schipper et al. 2008), which are declining due to environmental changes, impacts of anthropogenic pressure on wildlife habitats, and poaching (Maisels et al. 2013). Some of the species are completely protected under the schedule of the Wildlife Protection Act of 1972. There are many species which are highly endangered with only single populations in the entire distribution range, for example, the Kashmir stag or hangul (*Cervus elaphus hanglu*), the Manipur brow-antlered deer or sangai (*Cervus eldi eldi*), the Central Indian race of the swamp deer or barasingha (*Cervus duvauceli branderi*) and the Indian wild ass or khur (*Equus hemionus khur*) (Daniel 1991). *Cervus elaphus wallichi* has disappeared from Sikkim (Sankar and Goyal 2004). The decline of these populations of ungulates to adapt to environmental changes decreases their chances of long-term survival. Terrestrial mammals are threatened to the risk of extinction due to hunting pressure, habitat fragmentation, and habitat modification (Karanth et al. 2010), and around 50% of them are showing a declining trend in the population size from their native range (Channell and Lomolino 2000; Ceballos et al. 2005). Hence, these populations need a higher priority of conservation. As for other wildlife species of India, they are facing severe threats due to alarming increase in the human population (Karanth et al. 2009). Conservation success largely depends upon identifying vulnerable species and understanding the environmental factors that support their persistence in human-dominated landscapes (Kumar et al. 2017). More recently, genetic comparisons with the non-invasive sampling have led to greater understanding of lineages of related species, especially at higher taxonomic levels, where derived morphological characteristics can be difficult to determine owing to ancient divergences, thus leading to often radically different phylogenies and species groupings (Waits and Paetkau 2005).

The identification of species with non-invasive sampling without disturbing the animals or putting them at health risk still stands as one of the most basic but important issues in a forest. In a recent study, molecular taxonomy has helped in resolving the phylogeny of cervids resulting in clarity on species distribution and relatedness for effective conservation planning (Gilbert et al. 2006). However, the studies indicate further revision in the molecular phylogeny (Groves and Grubb 2011). Successful conservation efforts depend upon the identification of evolutionary significant units (ESU) of vulnerable species. In the present study, we examined *COI* and *Cytb* diversity within and among 22 species of Indian ungulates with the goal of testing the utility of DNA barcoding as a tool to identify species. The mitochondrial DNA (mtDNA) cytochrome c oxidase I (*COI*) and cytbchrome b (*Cytb*) has been widely used as a barcode for biological identification and phylogenetic studies (Hebert et al. 2003). In this study, we determine levels of interspecific variation within *COI* and *Cytb* between closely related species and provide an unbiased analysis using the same criteria for each and will make recommendations based on their use in phylogenetic reconstruction and species discrimination in between the 22 ungulates of India from the DNA extracted from noninvasive and highly degraded samples.

## Materials and methods

A total of 83 fresh fecal samples were collected from different protected and local areas of Tamil Nadu and Telangana. The permission was obtained from Chief Wildlife Warden [Ref no of letter WL5(A)/22918 and PCCF(WL)/E2/CR-17/2018-19]. A total of 14 species of Indian ungulates samples were collected from Arignar Anna Zoological Park in Chennai, Telangana and Nehru Zoological Park in Hyderabad (Table 1). All samples were fixed in 95% ethanol and stored at 20 °C until the analysis. A downloaded sequence of eight species from NCBI is also included in this study. A total of 22 species of Indian ungulates were included in the present study (Table 1). Genomic DNA was extracted from fresh fecal samples, by using QIAamp DNA Stool (QIAGEN, Hilden, Germany) with a little modification in temperature. A partial fragment of the *COI-1* gene was amplified using the following primers: *COI* (F_2_-GTACCGCTAATAATTGGTGCTCC), *COI* (R_2_-GGGTGGCCAAAGAATCAGAACAAGTG) (Kumar et al. 2017), *Cytb* Bongo forward 5′-GAT ACGTCCTACCATGAGGACAAATAT-3′, and *Cytb* Bongo reverse 5′-GGGTGTATTAAGTGGGTTTG-3′ (Faria et al. 2011). PCR amplification of the *COI* and *Cytb* gene was performed in a total volume of 25 µL reaction, containing 1X PCR Buffer (5 mM MgCl_2_; 10 mM dNTPs; 5 pmol of each primer; 1 U Taq polymerase (CinnaGen)). Negative controls were included in all PCR amplification. PCR reactions were carried out in Eppendorf Thermo Cycler and amplification conditions were 94 °C for 5 min followed by 35 cycles at 94 °C for 30 s, annealing 50 °C (T_a_) for 30 s and 72 °C for 1 min, with the final extension of 72 °C for 10 min. PCR products, that yielded a clear band on agarose gel electrophoresis, were used for sequencing bidirectionally, using an automated capillary sequencer (ABI377) following the manufacturer’s instructions.

**Table 1.** Species, location of collected fecal pellets of ungulates and sequences submitted and downloaded from NCBI.

Species	Common name	Collection site	Location (lat. & log.)	Genbank accession number (*COI*)	Genbank accession number (*Cytb*)
*Antilope cervicapra*	Blackbuck	Chennai Zoo, Tamil Nadu	12.8793°N, 80.0819°E	MH817002–MH817008, MK393407–MK393408	MN125149–MN125154, MN125156–MN125157
		Guindy National Park, Tamil Nadu	13.0049°N, 80.2379°E	MH817009–MH817013, MK393410–MK393414	MN125148, MN125155, MN125158
*Cervus duvaucelii*	Barasingha	Chennai Zoo, Tamil Nadu	12.8793°N, 80.0819°E	MK393415–MK393420	MN125162–MN125166
*Cervus unicolor*	Sambar	Chennai Zoo, Tamil Nadu	12.8793°N, 80.0819°E	MK393424–MK393425	MN816378
*Muntiacus muntjak*	Barking deer	Chennai Zoo, Tamil Nadu	12.8793°N, 80.0819°E	MK393422, MH817014–MH817016	MN125159–MN125161
*Axis axis*	Chital	Guindy National Park, Tamil Nadu	13.0049°N, 80.2379°E	MN481553–MN481559	MN792860–MN792866
*Ovis aries*	Domestic sheep	Maduari, Tamil Nadu	9.9252°N, 78.1198°E	MN102715, MN537880–MN537881	MN756681–MN756685
*Capra hircus*	Domestic goat	Maduari, Tamil Nadu	9.9252°N, 78.1198°E	MN102709–MN102712	MN125179–MN125183
*Bos gaurus*	Indian bison	Chennai Zoo, Tamil Nadu	12.8793°N, 80.0819°E	MK393426–MK393428	MN792857–MN792859
		Hyderabad Zoo, Telangana	17.3507°N, 78.4513°E	MN102713	
*Bubalus bubalis*	Domestic buffalo	Maduari, Tamil Nadu	9.9252°N, 78.1198°E	MN481546–MN481547	MN756686–MN756688
*Bos indicus*	Indian cow	Maduari,Tamil Nadu	9.9252°N, 78.1198°E	MN102705–MN102708, MN481560–MN481561	MN756691–MN756695
*Boselaphus tragocamelus*	Nilgai	Chennai Zoo, Tamil Nadu	12.8793°N, 80.0819°E	MN10270814, MK393429–MK393431, MN481545	MN125167–MN125169
*Moschiola indica*	Mouse Deer	Hyderabad Zoo, Telangana	17.3507°N, 78.4513°E	MN481548–MN481549	MN756689–MN756690
*Sus scrofa*	Wild boar	Mahavir Wild Life Sanctuary	17.3662°N, 78.6282°E	MN481550–MN481552	MN792867–MN792869
Downloaded from NCBI					
*Tetracerus quadricornis*	Four-horned antelope			KT372097, NC_020788, EF536355	AF036274, NC_020788, EF536355
*Rhinoceros unicornis*	Indian rhinoceros			JN417004	JF718877, NC_001779
*Bos grunniens*	Domestic yak			HQ269432, KX859289, HQ269464–HQ269466	KR676428–KR676431
*Equus caballus*	Domestic horse			JN228963, JQ735458–JQ735459	EU433684–EU433686, JF718882
*Porcula salvania*	Pygmy hog			NC_043879, MN095549	NC_043879, MN095549, EU107788
*Axis porcinus*	Indian hog deer			MH443786–MH443790, KT372095	DQ379301, EU878394, FJ556572, FJ556558
*Muntiacus putaoensis*	Leaf deer			NC_036430, MF737190	MF737181, MF737180, MF737179
*Rucervus duvaucelii branderi*	hard ground barasingha			MG744445, NC_039091, MG788693, MG770614	MG744445, NC_045060, MG770614, MG788693
*Gazella bennettii*	Chinkara			KT372099	JN410357, JN410341, JN410340, NC_020703

## Data analysis

All the sequences were individually checked manually using the program BioEdit and ClustalW (http://www.clustal.org/clustal2/). Each sequence was systematically analyzed to find out the identity through Basic Local Alignment Search Tool (BLASTn; https://blast.ncbi.nlm.nih.gov). All the sequences obtained were submitted to NCBI to obtain the respective accession numbers. We have retrieved sequences of three species from GenBank from the whole mitochondrial genome. Alignments were then performed using BioEdit (Hall 1999) and ClustalW (http://www.clustal.org/clustal2/) trimmed to 408 bp. All statistical parameters, sequence composition and substitution pattern for the entire data set, genetic divergence, variable sites, transition, and transversion rates were calculated using the program MEGA 6 (Tamura et al. 2013). The Bayesian tree was built in Mr. Bayes 3.1.233, the program Modeltest was used to find the suitable model for data test by selecting parameters nst = 6 for GTR + G + I model with four metropolis-coupled Markov Chain Monte Carlo (MCMC) and run for 1,000,000 cycles with 25 burns (Ronquist and Huelsenbeck 2003). The generated BA tree was represented by the FIGTree software. The neighbor-joining (NJ), maximum-parsimony (MP), and maximum-likelihood (ML) were also generated by MEGA 6 (Tamura et al. 2013). The haplotype data were generated using DnaSP5.10 (Librado and Rozas 2009). Automatic barcode gap discovery analysis (ABGD) was implemented online (www.abi.snv.jussieu.fr/public/abgd/abgdweb.html, Puillandre et al. 2012) and was run by selecting Kimura 2-parameter distance (K2P) with transition/transversion ratio (TS/TV) equal to 2 and with a FASTA file input of the alignment, with default values for *P*_min_, *P*_max_, and relative gap width. The database sequence of *Pteropus giganteus* (MG821199) was used as an out-group in the phylogenetic study for making the Bayesian tree.

## Results and discussion

A total number of 83 fecal samples out of these 67 samples obtained good amplification in *COI* and 65 in *Cytb* gene, the other samples might have yielded low DNA or were highly degraded samples. Most of the sequences generated 470 bp in *COI* and 420 bp but trimmed to 408 bp, as few shorter sequences were downloaded from NCBI, in both *COI* and *Cytb*. The generated DNA sequences of 14 species of Indian ungulates were submitted to NCBI with accession numbers given in Table 1. *COI* genes of five species, *Axis porcinus, Rucervus duvacelii branderi, Muntiacus putaonesis, Tetracerus quadricornis*, and *Porcula salvania*, that were retrieved from the whole mitochondrial genome, were not obtained from NCBI. A total of 93 sequences of *COI* and 86 of *Cytb* of 22 species were included in this study. The partial region of 408 bp of *COI* gene was analyzed, out of these 212 bp (51.9%) were conserved, 196 (48.0%) variable, 31 (7.6%) singleton, and 165 (40.4%) were parsimony informative. Overall 48 haplotypes were observed from 22 species of ungulates in *COI*. The overall haplotype diversity was 0.956 and nucleotide diversity was 0. 1483.

In *Cytb*, 205 (50.2%) of 408 sites varied among taxa, 183 (44.8%) parsimony-informative, 203(49.7%) conserved and 22 (5.3) singleton. Overall, 45 haplotypes were observed in *Cytb*. The overall haplotype diversity was 0.974 and nucleotide diversity was 0.1529. The combined sequences of the two gene segments had 816 sites, of which 350 (42.9%) were parsimony informative.

With respect to the pairwise distance among the 22 ungulates species, the highest interspecific genetic divergence observed was 0.295 (29.5%) between *Equus caballus* and *Ovis aries* in *COI* and 0.288 (28.8%) between *E. caballus* and *Moschiola indica* in *Cytb* and the lowest genetic divergence was 0.022 (2.2%) in *COI* and 0.023 (2.3%) in *Cytb* in *R. d. branderi* and *R. duvaucelii* (Table 2). The overall mean divergence was estimated at 17.2% in both *COI* and *Cytb*. The highest intraspecific variation was observed in *O. aries* (3.9%) and lowest (0.01) in *Antilope cervicapra*, *Axis axis*, and *Muntiacus muntjak* (Table 2) in *COI*. In the *Cytb* gene, the highest intraspecific sequence divergence was 0.137 (*P. salvania*) and the lowest 0.001 (*O. aries*, *Bos grunniens*, *A. cervicapra*) (Table 2).

**Table 2.** Pair wise genetic distance using the Kimura 2-parameter model (K2P) between *COI* (below diagonal) and the *Cytb* genes (above diagonal) of 22 Indian ungulates.

	*Antilope cervicapra*	*Gazella Bennettii*	*Rucervus duvacelii branderi*	*Rucervus duvaucelii*	*Cervus unicolor*	*Muntiacus putaoensis*	*Muntiacus muntjak*	*Axis porcinus*	*Axis axis*	*Ovis aries*	*Capra hircus*	*Bos gaurus*	*Bos indicus*	*Bubalus bubalis*	*Boselaphus tragocamelus*	*Tetracerus quadricornis*	*Moschiola indica*	*Rhinoceros unicornis*	*Porcula salvania*	*Sus scrofa*	*Equus caballus*	*Bos grunniens*
*Antilope cervicapra*	**0.001/ 0.001**	0.102	0.187	0.163	0.173	0.162	0.165	0.192	0.141	0.149	0.158	0.148	0.182	0.167	0.135	0.154	0.196	0.232	0.220	0.217	0.240	0.155
*Gazella Bennettii*	0.085	**0.000/ 0.019**	0.178	0.178	0.193	0.176	0.193	0.186	0.187	0.156	0.180	0.161	0.181	0.154	0.145	0.150	0.196	0.228	0.221	0.211	0.233	0.159
*Rucervus duvacelii branderi*	0.205	0.195	**0.009/ 0.000**	0.023	0.104	0.156	0.153	0.099	0.103	0.149	0.181	0.194	0.193	0.188	0.163	0.167	0.248	0.276	0.256	0.256	0.260	0.183
*Rucervus duvaucelii*	0.191	0.179	0.022	**0.001/ 0.000**	0.095	0.143	0.141	0.096	0.088	0.140	0.157	0.177	0.189	0.185	0.147	0.150	0.248	0.276	0.255	0.246	0.249	0.179
*Cervus unicolor*	0.163	0.166	0.102	0.089	**0.000/ 0.000**	0.152	0.153	0.119	0.118	0.169	0.176	0.199	0.226	0.212	0.189	0.197	0.254	0.268	0.261	0.242	0.243	0.205
*Muntiacus putaoensis*	0.173	0.153	0.115	0.115	0.112	**0.000/ 0.002**	0.068	0.134	0.138	0.179	0.145	0.165	0.207	0.181	0.163	0.180	0.233	0.258	0.254	0.247	0.284	0.171
*Muntiacus muntjak*	0.154	0.164	0.121	0.107	0.112	0.052	**0.001/ 0.002**	0.143	0.126	0.161	0.163	0.184	0.224	0.203	0.176	0.214	0.226	0.226	0.281	0.256	0.287	0.196
*Axis porcinus*	0.187	0.181	0.067	0.070	0.095	0.099	0.114	**0.004/ 0.064**	0.099	0.165	0.182	0.194	0.199	0.176	0.172	0.172	0.248	0.283	0.256	0.239	0.266	0.168
*Axis axis*	0.207	0.193	0.070	0.075	0.107	0.116	0.123	0.051	**0.001/ 0.000**	0.143	0.188	0.190	0.210	0.198	0.166	0.196	0.226	0.269	0.256	0.253	0.275	0.190
*Ovis aries*	0.172	0.187	0.212	0.201	0.202	0.168	0.189	0.208	0.234	**0.039/ 0.001**	0.121	0.192	0.189	0.152	0.151	0.168	0.224	0.223	0.227	0.234	0.234	0.175
*Capra hircus*	0.169	0.174	0.201	0.185	0.199	0.163	0.185	0.187	0.199	0.111	**0.010/ 0.004**	0.153	0.195	0.154	0.131	0.139	0.235	0.249	0.232	0.213	0.242	0.156
*Bos gaurus*	0.184	0.180	0.209	0.192	0.195	0.190	0.183	0.221	0.238	0.226	0.199	**0.000/ 0.007**	0.101	0.143	0.136	0.142	0.207	0.273	0.240	0.246	0.251	0.090
*Bos indicus*	0.181	0.170	0.196	0.178	0.187	0.169	0.168	0.213	0.212	0.210	0.203	0.082	**0.026/ 0.062**	0.143	0.144	0.149	0.212	0.267	0.250	0.256	0.264	0.086
*Bubalus bubalis*	0.150	0.156	0.201	0.199	0.187	0.174	0.175	0.188	0.199	0.216	0.192	0.164	0.168	**0.028/ 0.000**	0.139	0.132	0.230	0.213	0.211	0.216	0.223	0.112
*Boselaphus tragocamelus*	0.195	0.170	0.200	0.184	0.178	0.147	0.155	0.186	0.175	0.196	0.165	0.189	0.179	0.172	**0.010/ 0.000**	0.083	0.083	0.244	0.212	0.199	0.241	0.132
*Tetracerus quadricornis*	0.181	0.165	0.175	0.166	0.134	0.153	0.171	0.158	0.174	0.161	0.147	0.206	0.196	0.134	0.139	**0.008/ 0.000**	0.206	0.233	0.200	0.204	0.225	0.148
*Moschiola indica*	0.188	0.198	0.223	0.218	0.194	0.206	0.191	0.206	0.218	0.240	0.243	0.246	0.210	0.214	0.211	0.212	**0.028/ 0.000**	0.262	0.231	0.247	0.288	0.214
*Rhinoceros unicornis*	0.244	0.224	0.248	0.244	0.248	0.206	0.216	0.254	0.248	0.273	0.242	0.235	0.217	0.250	0.219	0.243	0.227	**0.000/ 0.000**	0.248	0.241	0.199	0.241
*Porcula salvania*	0.245	0.256	0.225	0.215	0.268	0.211	0.218	0.229	0.242	0.219	0.225	0.252	0.213	0.230	0.215	0.247	0.262	0.245	**0.000/ 0.137**	0.149	0.247	0.225
*Sus scrofa*	0.211	0.244	0.240	0.237	0.254	0.237	0.249	0.247	0.263	0.217	0.218	0.262	0.235	0.220	0.246	0.232	0.237	0.264	0.107	**0.012/ 0.024**	0.248	0.235
*Equus caballus*	0.242	0.231	0.268	0.248	0.278	0.269	0.245	0.269	0.289	0.295	0.239	0.247	0.248	0.275	0.245	0.270	0.240	0.221	0.263	0.273	**0.025/ 0.002**	0.242
*Bos grunniens*	0.162	0.160	0.195	0.182	0.192	0.188	0.184	0.208	0.206	0.216	0.210	0.065	0.072	0.144	0.163	0.176	0.203	0.224	0.226	0.233	0.238	**0.003/ 0.001**

The mean genetic distance with in species are represented in bold numbers on diagonal (*COI*/*Cytb*).

**Figure 1.**
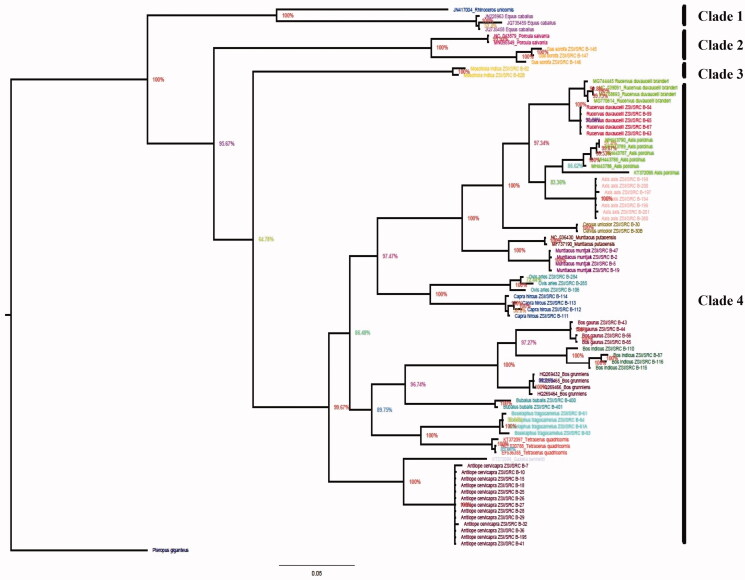
The Bayesian tree from combined analysis of the *Cytb* and *COI* sequences of Indian ungulates and well-supported Clade 1 to 4 (posterior probability values are shown at each node).

The estimated ABGD analysis of *COI* (Figure 1) revealed a total 22 MOTUs within the studied barcode data in the dataset. One of the species *Bos indicus* showed similar results in ABGD analysis and BA tree topology showing two MOTUs with two sister’s clades. However, the other two species depicted inconsistent results in ABGD analysis, *R. d. branderi* is the subspecies of *R. duvacelii* (Groves and Grubb 2011) but in ABGD analysis results, *R. duvacelii* and *R. d. branderi* were considered a single species. This could be confirmed by many more markers.

**Figure 2.**
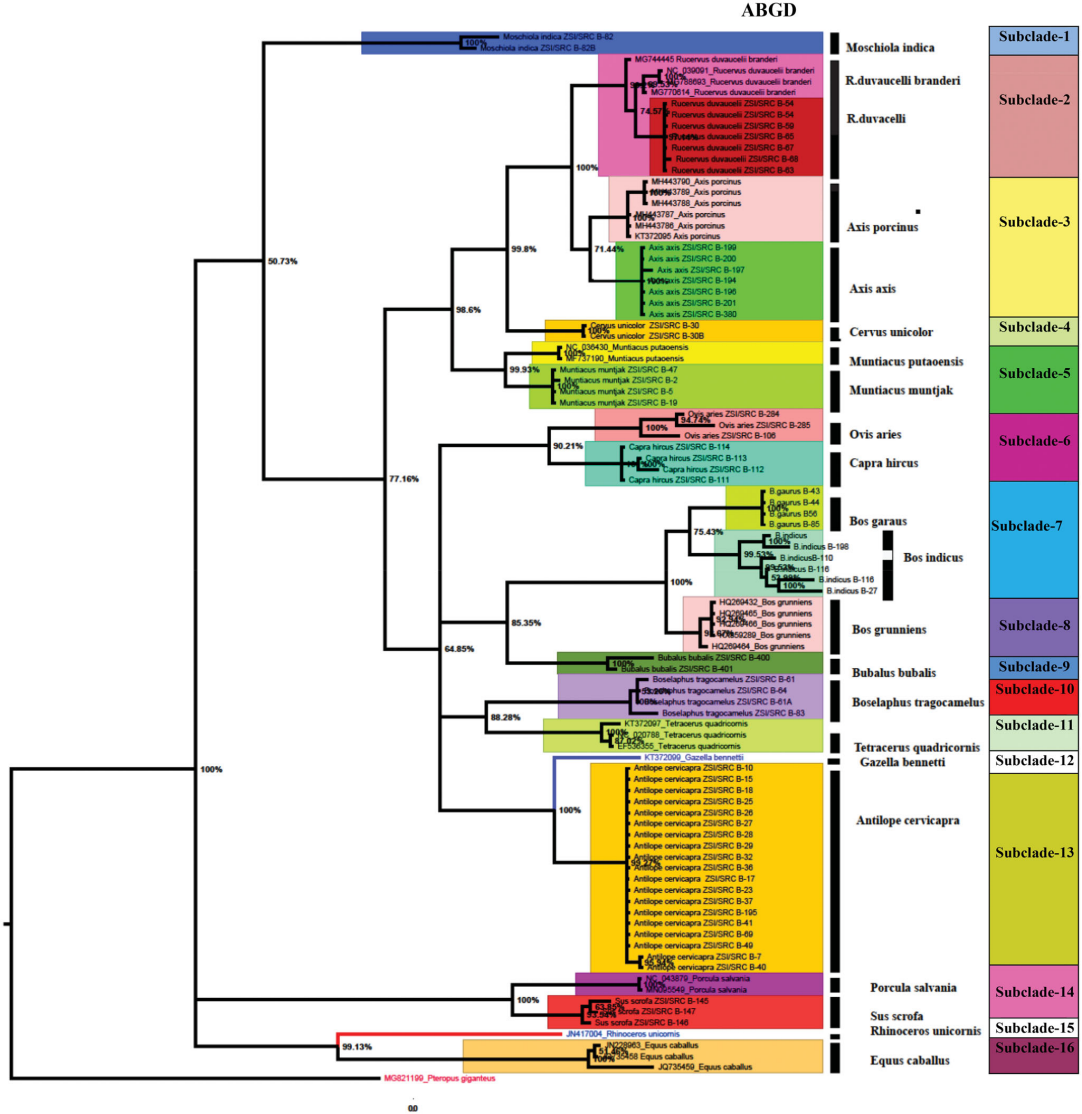
The Bayesian analysis tree showing the multiple clades and paraphyletic clustering of Indian ungulates with both generated and database sequences. Species delimitation through ABGD analysis is denoted by black bar beside each clade. Sixteen subclades are represented by different color bars.

The topology patterns are almost alike in all the tree-building methods (NJ, ML, and BA) examined for the studied dataset of 22 species of Indian ungulates with high bootstrap values in *COI* and combined sequences of both genes *COI* and *Cytb* (Figures 1 and 2). The bayesian tree is produced by combined sequences of two genes falling into four major clades. *Rhinoceros unicornis* and *E. caballus* are clustered in Clade 1. In Clade 2, *P. salvania* is close to *Sus scrofa*. *Moschiola indica* alone is separated in Clade 3. Clade 4 comprises the family Bovidae and Cervidae (Figure 1). In a separate analysis of *COI*, there are 16 sub-clades identifying the species in the entire tree with 22 distinct lineages representing all the 22 separate species in *COI* (Figure 2). Sub clade 1: *Moschiola indica*; sub clade 14: *S. scrofa*; Sub clade 15: *R. unicornis*; and Sub clade 16: *E. caballus* are separated as paraphylitic group from all the ungulates species (Figure 2). The rhino (*R. unicornis*) is closer to horse (*E. caballus*). Pygmy hog (*P. salvania*) and wild boar (*S. scrofa*) population are sisters to each other. Recent findings of a genomic analysis on pygmy hog reveal extensive interbreeding of wild boar (Liu et al. 2019). Indian spotted deer (*A. axis*) and hog deer populations are clustered together as sister species in clade 3 but paraphyletic with swamp and Sambar deer. The clade two suggested two subspecies of swamp deer population. A similar finding was reported by (Kumar et al. 2017). *Rucervus duvacelii branderi* is the subspecies of *R. duvacelii* (Groves and Grubb 2011) but the genetic distance is low (0.023) (Table 2). We found three nucleotide deletions in *R. duvaucelii* compared to *R. d. branderi*. Both species are separated by a high bootstrap value (98%) (Figure 2). The Clade 11, four-horned antelope *T. quadricornis* is the sole member of the genus *Tetracerus*, and is placed under the family Bovidae is clustered with the nilgai (*Boselaphus tragocamelus*) in the Boselaphini, (Leslie and Sharma 2009). *Tetracerus quadricornis* and *B. tragocamelus* (Nilgai) are clustered together as a monophyletic group and this cluster is again paraphyletic cluster with genus *Bos* in Bovidae family. *Antilope cervicapra* and *Gazella bennettii* form a paraphyletic group closer to (sheep) *O. aries* and *Capra hircus* (goat) in Bovidae family. Our study compared barcode data of *COI* and *Cytb* in Indian ungulates and may serve as a baseline for future analyses of genetic diversity of ungulates (Ramon-Laca et al. 2014).

Therefore, the present study provides significant contributions toward the taxonomic identity confirmation, phylogenetic studies that can be used for better planning of conservation and management of Indian ungulates. In India, very few data are present on many species of ungulates. This study will be helpful to strengthen the global database with barcode sequences of accurately identified other mammalian species from Fecal DNA. Thus, the improvements of both taxonomic studies, generated barcode data are mandatory for more reliable and accurate results. The DNA sequences of *COI* and *Cytb* genes revealed that the obtained sequences are very helpful to delineate the Indian ungulates (Bergsten et al. 2012).

## References

[CIT0001] Bergsten J, Bilton DT, Fujisawa T, Elliott M, Monaghan MT, Balke M, Hendrich L, Geijer J, Herrmann J, Foster GN, et al. 2012. The effect of geographical scale of sampling on DNA barcoding. Syst Biol. 61(5):851–869. 22398121 10.1093/sysbio/sys037 PMC3417044

[CIT0002] Ceballos G, Ehrlich PR, Soberón J, Salazar I, Fay JP. 2005. Global mammal conservation: what must we manage? Science. 309(5734):603–607. 16040704 10.1126/science.1114015

[CIT0003] Channell R, Lomolino MV. 2000. Dynamic biogeography and conservation of endangered species. Nature. 403(6765):84–86. 10638757 10.1038/47487

[CIT0004] Daniel CJ. 1991. Ungulate conservation in India – problems and prospects. Appl Animal Behav Sci. 29(1–4):349–359.

[CIT0005] Faria PJ, Kavembe GD, Jung’a JO, Kimwele CN, Estes LD, Reillo PR, Mwangi AG, Bruford MW. 2011. The use of non-invasive molecular techniques to confirm the presence of mountain bongo *Tragelaphus eurycerus isaaci* populations in Kenya and preliminary inference of their mitochondrial genetic variation. Conserv Genet. 12(3):745–751.

[CIT0006] Gilbert C, Ropiquet A, Hassanin A. 2006. Mitochondrial and nuclear phylogenies of Cervidae (Mammalia, Ruminantia): systematics, morphology and biogeography. Mol Phylogenet Evol. 40(1):101–117. 16584894 10.1016/j.ympev.2006.02.017

[CIT0007] Groves C, Grubb P. 2011. Ungulate taxonomy. Baltimore (MD): Johns Hopkins University Press; p. 317.

[CIT0008] Hall TA. 1999. BioEdit: a user‐friendly biological sequence alignment editor and analysis program for Windows 95/98/NT. Nucleic Acids Symposium Series. 41:95–98.

[CIT0009] Hebert PDN, Cywinska A, Ball SL, deWaard JR. 2003. Biological identifications through DNA barcodes. Proc R Soc Lond B. 270(1512):313–321. 10.1098/rspb.2002.2218 PMC1691236 12614582

[CIT0010] Karanth KK, Nichols JD, Hines JE, Karanth KU, Christensen NL. 2009. Patterns and determinants of mammal species occurrence in India. J Appl Ecol. 46:1189–1200.

[CIT0011] Karanth KK, Nichols JD, Karanth KU, Hines JE, Christensen NL. 2010. The shrinking ark: patterns of large mammal extinctions in India. Proc R Soc B. 277(1690):1971–1979. 10.1098/rspb.2010.0171 PMC2880105 20219736

[CIT0012] Kumar A, Ghazi MGU, Singh B, Hussain SA, Bhatt D, Gupta SK. 2017. Conserve primers for sequencing complete ungulate mitochondrial cytochrome c oxidase I (*COI*) gene from problematic and decomposed biological samples. Mitochondrial DNA Part B. 2(1):64–66. 33490438 10.1080/23802359.2016.1247672 PMC7800399

[CIT0013] Leslie DM, Sharma K. 2009. *Tetracerus quadricornis* (Artiodactyla: Bovidae). Mammalian Species. 843:1–11.

[CIT0014] Librado P, Rozas J. 2009. DnaSP v5: a software for comprehensive analysis of DNA polymorphism data. Bioinformatics. 25(11):1451–1452. 19346325 10.1093/bioinformatics/btp187

[CIT0015] Liu L, Bosse M, Megens HJ, Frantz LAF, Lee YL, Evan K, Pease EKI, Narayan G, Groenen MAM, Madsen O. 2019. Genomic analysis on pygmy hog reveals extensive interbreeding during wild boar expansion. Nat Commun. 10(1):1992. 31040280 10.1038/s41467-019-10017-2 PMC6491599

[CIT0025] Maisels F, Strindberg S, Blake S. et al. 2013. Devastating decline of forest elephants in Central Africa. PLoS ONE. 8(3):e59469. 23469289 10.1371/journal.pone.0059469 PMC3587600

[CIT0016] Puillandre N, Lambert A, Brouillet S, Achaz G. 2012. ABGD, automatic barcode gap discovery for primary species delimitation. Mol Ecol. 21(8):1864–1877. 21883587 10.1111/j.1365-294X.2011.05239.x

[CIT0017] Ramon-Laca NA, Gleeson D, Yockney I, Perry M, Nugent G. 2014. Reliable discrimination of 10 ungulate species using high resolution melting analysis of faecal DNA. PLoS ONE. 9(3):e92043. 24637802 10.1371/journal.pone.0092043 PMC3956866

[CIT0018] Ronquist F, Huelsenbeck JP. 2003. MrBayes 3: Bayesian phylogenetic inference under mixed models. Bioinformatics. 19(12):1572–1574. 12912839 10.1093/bioinformatics/btg180

[CIT0019] Sankar K, Goyal SP. 2004. Ungulates of India. ENVIS bulletin: wildlife and protected areas, Vol. 07, No. 1. Dehradun, India: Wildlife Institute of India.

[CIT0020] Schipper J, Chanson JS, Chiozza F, Cox NA, Hoffmann M, Katariya V, Lamoreux J, Rodrigues ASL, Stuart SN, Temple HJ, et al. 2008. The status of the world’s land and marine mammals: diversity, threat and knowledge. Science. 322(5899):225–230. 18845749 10.1126/science.1165115

[CIT0021] Tamura K, Stecher G, Peterson D, Filipski A, Kumar S. 2013. MEGA6: Molecular Evolutionary Genetics Analysis software version 6.0. Mol Biol Evol. 30(12):2725–2729. 24132122 10.1093/molbev/mst197 PMC3840312

[CIT0022] Waits LP, Paetkau D. 2005. Noninvasive genetic sampling tools for wildlife biologists: a review of applications and recommendations for accurate data collection. J Wildl Manag. 69(4):1419–1433.

